# Using Patient Flow Information to Determine Risk of Hospital Presentation: Protocol for a Proof-of-Concept Study

**DOI:** 10.2196/resprot.5894

**Published:** 2016-12-20

**Authors:** Christopher M Pearce, Adam McLeod, Jon Patrick, Douglas Boyle, Marianne Shearer, Paula Eustace, Mary Catherine Pearce

**Affiliations:** ^1^ Melbourne East General Practice Network Burwood East Australia; ^2^ Health Language Analytics Eveleigh Australia; ^3^ Research Information Technology Unit Health and Biomedical Informatics Centre The University of Melbourne Parkville Australia; ^4^ Gippsland Primary Health Network Moe Australia; ^5^ Eastern Melbourne Primary Health Network Box Hill Australia

**Keywords:** electronic health record, data linkage, primary care, machine learning, avoidable presentation

## Abstract

**Background:**

Every day, patients are admitted to the hospital with conditions that could have been effectively managed in the primary care sector. These admissions are expensive and in many cases are possible to avoid if early intervention occurs. General practitioners are in the best position to identify those at risk of imminent hospital presentation and admission; however, it is not always possible for all the factors to be considered. A lack of shared information contributes significantly to the challenge of understanding a patient’s full medical history. Some health care systems around the world use algorithms to analyze patient data in order to predict events such as emergency presentation; however, those responsible for the design and use of such systems readily admit that the algorithms can only be used to assess the populations used to design the algorithm in the first place. The United Kingdom health care system has contributed data toward algorithm development, which is possible through the unified health care system in place there. The lack of unified patient records in Australia has made building an algorithm for local use a significant challenge.

**Objective:**

Our objective is to use linked patient records to track patient flow through primary and secondary health care in order to develop a tool that can be applied in real time at the general practice level. This algorithm will allow the generation of reports for general practitioners that indicate the relative risk of patients presenting to an emergency department.

**Methods:**

A previously designed tool was used to deidentify the general practice and hospital records of approximately 100,000 patients. Records were pooled for patients who had attended emergency departments within the Eastern Health Network of hospitals and general practices within the Eastern Health Network catchment. The next phase will involve development of a model using a predictive analytic machine learning algorithm. The model will be developed iteratively, testing the combination of variables that will provide the best predictive model.

**Results:**

Records of approximately 97,000 patients who have attended both a general practice and an emergency department have been identified within the database. These records are currently being used to develop the predictive model.

**Conclusions:**

Records from general practice and emergency department visits have been identified and pooled for development of the algorithm. The next phase in the project will see validation and live testing of the algorithm in a practice setting. The algorithm will underpin a clinical decision support tool for general practitioners which will be tested for face validity in this initial study into its efficacy.

## Introduction

### Overview

Primary care management of patients makes good economic sense. When compared to the high-cost, high-intensity activities of secondary or tertiary care systems [1,2], primary care provides a low-cost, low-intensity approach to health care that is ideally suited to addressing both illness management and prevention [2,3].

Many people have multiple risk factors for more than one health problem, and it is well recognized that the risk effect is magnified when risk factors are combined. Reducing and minimizing risks through attention to preventative measures, timely intervention, and optimal use of support strategies and services can minimize the risk of harm and encourage better use of hospital resources. Missing preventative opportunities, on the other hand, can result in a higher dependency on limited and expensive hospital resources.

Although many conditions cannot be prevented and will at some point require management in a hospital, a great number will be well managed at home, in general practice, or by community-based services. These ambulatory care–sensitive conditions (ACSCs) are conditions for which management is influenced by primary care access, care funding, and the patient’s socioeconomic status, as well as the medical condition itself [4]. More vulnerable groups within the community, such as elderly patients [5], Aboriginal and Torres Strait Islanders [6], and patients of low health literacy [7], are considered more likely to present for emergency care. Both incentivizing care in order to lower direct primary care costs to patients and improving the management of such conditions in primary care may affect hospital admissions for those conditions [8].

In Australia, programs such as the Hospital Admission Risk Program (HARP) model in Victoria have specifically targeted patients at high risk of emergency department presentation [9]. Selection for these programs has been based on the number of admissions or presentations or the chronic disease—predominantly chronic obstructive pulmonary disease, congestive heart failure, and diabetes. There is, however, an inherent difficulty in obtaining all the relevant information when considering access to such programs because the large number of variables is beyond what can be documented and widely understood in the set of admission criteria. For example, in any clinical interaction, the recorded data is only a subset of the actual data. By using a computerized clinical decision support system (CCDSS) to assess all available historical data rather than simply the most recently collected data, we hope to improve the decision-making process. There is evidence to suggest such systems can improve chronic disease management and in some cases patient outcomes as well; however, further work is required to fully understand the limitations of the CCDSSs that are currently available and in use. The key to developing a good system will certainly lie with developing a good predictive algorithm or model [10].

Several admission prediction models have been implemented in the United Kingdom, where a unified health system allows for unified patient flows [11]. One of the most widely reported models was the Patients at Risk of Rehospitalization (PARR++) Combined Predictive Model [12]. This model, like many other models of this kind, primarily used hospital data for predicting risk of emergency department (ED) readmission and proved relatively ineffective in changing clinical outcomes. A more complex model, involving over 30 variables, is based on general practice data alone [13]. Other approaches have taken a disease-specific view rather than a whole of population view [14]. In Australia and other countries with nonunified health care systems, isolated datasets have been used to build similar models. One such model, the Patient Admission Prediction Tool, has been implemented in Queensland, Australia, in order to improve bed management in hospitals where demand is ever increasing [15,16]. This model used hospital admission data to forecast future ED presentation; however there was no link to diagnosis. While the tool was deemed successful at forecasting attendance, very little impact was made on the key markers of hospital overcrowding, a failing that highlights the importance of preventing the hospital attendance in the first place.

A possible solution to identifying the potential ED presentation in general practice involves using prehospital presentation markers from general practice attendances. In order to reduce the number of preventable ED presentations, data linkage models generated [17] across health care settings are needed. The problem is that delivering data in a real-time mode to the point-of-care in order to most effectively influence care remains a significant challenge.

The Population Level Analysis and Reporting (POLAR) diversion project will set up the facility to test the hypothesis that risk reduction for multiple patient demographics and conditions can be achieved in the Australian context through strategic syntheses and intersects of extracted clinical data. This will build on ontological work conducted in the Australian context to more reliably flag conditions associated with increased hospital admission, such as diabetes, from routine data [18].

In doing so it attempts to address a significant gap in suitable strategies currently available, which are aimed at identifying avoidable ED presentations. The process aims to add a further depth and breadth to the clinical decision aids available at point-of-care. It particularly aims to facilitate the preventative orientations called for by best practice approaches [4,17], focusing the attention of busy general practitioners on risk reduction over crisis management.

This proof-of-concept study develops and tests the risk prediction process. Development of this risk prediction tool will use data housed in a warehouse that feeds the POLAR tool, a resource for health professionals used to analyze and interpret health records. The ultimate outcome of this study will be to implement a predictive model in general practices aimed at reducing avoidable presentations to hospital EDs.

### Study Aims

#### Primary Aims

- Develop a predictive risk identification tool, which may be a risk tree or risk score
- Determine the validity of the data extraction/risk algorithm integration process by testing with a select number of practices
- Implement in the general practice environment to test the validity of the risk report
- Demonstrate the feasibility of a broader program roll-out and assess the general practitioner–defined interventions initiated in response to the risk reports

#### Secondary Aims

- Identify and construct ontologies that identify people with conditions, multimorbidity, and other risk factors (eg, economic disadvantage) associated with hospital admission
- Highlight gaps in data quality that might restrict the use of the predictive tool
- Identify decision-support strategies for use by general practices in maintaining and improving vigilance of patients with specified morbidities and comorbidities
- Improve timeliness of interventions in actual and potential complications
- Improve patient care at home and in the community
- Provide informed estimation of generated cost savings by costing analyses of resources used (at the general practice level) versus resources saved (at the hospital level)
- Support clinical governance in general practice

## Methods

### Setting

The Melbourne East General Practice Network (MEGPN) is a not-for-profit organization offering primary care services and supporting general practices in the area. It holds and manages the data warehouse that is integrated with the POLAR tool. Regular downloads are added from contributing general practices in the catchment, thus continually expanding the data pool. In a previous incarnation, MEGPN was funded by government to support general practices in the eastern suburbs of Melbourne, Australia’s second largest city [19]. For over 10 years, MEGPN has been offering practices quality improvement activities using the Plan/Do/Study/Act method. Central to the entire program has been MEGPN’s active encouragement through its practice feedback reporting of consistent data governance [20] and its independent data quality activities aimed at improving the data analysis used in the feedback visions [21].

### Ontologies for High Risk Conditions

We will develop clinical surrogates (eg, use medication data) and other markers that flag from routine data the risk of admission. This study will specifically examine general practice patients from the MEGPN region. There are currently 1.3 million deidentified records in the MEGPN general practitioner dataset, which includes many patients from outside the catchment. In Australia, patients are not bound to a specific practice or general practitioner and can visit any number of practices in a given time frame. Initial data on emergency presentations will be obtained from Eastern Health, the main provider of secondary care services to the region.

### Population

No distinction will be made with regard to any aspect of a patient’s medical history or demographics; any patient who meets the criteria in the algorithm will be highlighted to the general practice. The implementation phase evaluating the reporting process will use data from 6 to 10 practices from the pool that contributed to the research dataset.

### Ethics

As this is a multifaceted project, there are several aspects to the ethics applications involving various partner institutions and elements of the project. MEGPN has ethics approvals for the use of deidentified data in its database for the purposes of research and for reporting such data to general practice as well as additional approval for linking MEGPN data with deidentified hospital data. Separate ethics approval has been granted for a focus group interview informing selection of key algorithm components.

### Governance

The project has an advisory group consisting of general practitioners and hospital representatives and representatives from state and federal governments and the Australian Institute of Health and Welfare. The group provides an important validation mechanism and project advice around practitioner needs and clinical assumptions. The advisory group also assists in developing the specific alert criteria of the risk identification algorithm through a range of best practice clinical guidelines.

### Model Development

The first phase of the study involves understanding the general practice journeys of patients who attend the ED. To do this, data have been extracted from general practices in the area and linked with hospital emergency admission data. The hospital data has been collected from the Victorian Emergency Minimum Dataset (VEMD) where hospitals contribute all records for emergency admissions and includes demographic data, referral/arrival information, triage category, diagnosis and procedures, and discharge information.

In order to obtain the necessary granular general practice data, the project is implementing a data extraction tool. The Generic Health Network Information Technology for the Enterprise (GRHANITE) tool [22] extracts patient-centered data from the practices. The collected data include diagnoses (active and inactive), serial visit information, reason for encounter, procedures, referrals, pathology and diagnostic results, and comprehensive prescription information, as well as demographic data. Within GRHANITE is the ability to generate a unique encrypted hashtag linkage key to allow linking of individual patient data across sites. Both sets of data are therefore stripped of any identifying information but can be linked by the hashtag linkage key applied by the GRHANITE tool. The hospital data, which is episode-based, is then linked with the patient-centered data from general practice. We will therefore be able to detect those patients who have attended the local ED and any general practice in the area. The POLAR data warehouse holds ED data from over a 5-year period, and we will build a database of general practitioner attendances across all practices for the 6 months prior and 3 months after each admission.

A model will be developed using a predictive analytic machine learning algorithm. The modeling process will require us to build attribute sets around 14 groups of variables. Models will be built by omitting each attribute set to determine their effects on the models. They will then be evaluated by 10-fold cross validation on a support vector machine, identifying the precision and recall for each class. In an effort to create more refinement in the model, domains will be compacted where possible, most often to 3 values: below normal, above normal, and normal. This is a method for densifying the statistical sample and hopefully reinforcing weak effects. Some analysis will be performed using information gain to understand the level of contribution of each attribute set to the predictive model. Other exploration will be made with the number of classes that produced the most effective classification because classes for 60-day, 90-day, 180-day, and 365-day periods proved particularly difficult to model reliably.

Based on the model, an at-risk report will be created with these flags for the general practitioner: (1) patients deemed to be at heightened risk of increasing morbidity related to specific, targeted health states and (2) the parameters and thresholds exceeded that place them at current risk of presentation to the hospital. In order to continuously improve the quality of the report, we will request additional information from the general practitioner be provided that could enhance the accuracy of the predictive algorithm.

Upon completion of model development, consent will be obtained for validation by practices from their representatives, and individual general practitioners will be contracted for their evaluations in return for small incentive payments designed to cover their expenses in using the tool and providing feedback. Patients will be alerted that the practice is involved in the study, as per the responsible Human Research Ethics Committee requirements.

### Risk Score Implementation

Implementation of the risk report will be initiated in multiple practices that are already providing data to POLAR. Essentially, practices will have a regular data extraction that will be then run through the algorithm; the results will be uploaded to the practice in deidentified form for reidentification on a patient-by-patient basis by the practice software. The report is issued by internal identifier that can be cross-matched by practice staff to identify patient details within the practice. Thus patients can be identified only at the point-of-care. No identifying information will be kept centrally. General practitioners will be recruited to participate in focus groups and interviews on the impact on their personal practice of the algorithm-informed risk reporting process.

The risk report will serve as a clinical decision aid to be used with normative clinical discretion. It is not intended as either a clinical directive or a prescription for management. Rather, it flags for the general practitioner patients who meet at-risk criteria and reports on the parameters exceeded and parameter/morbidity combinations that trigger the alert. Data quality issues will also be raised with general practitioners, with the research team outlining missing information in the record (that if complete might mean better risk stratification).

During the study, practices will receive a series of reports showing estimated risk of ED presentation across the time periods 1 month, 3 months, and 12 months. General practitioners will be asked their thoughts on the clinical accuracy of the prediction against their clinical knowledge. This will be followed with a brief questionnaire asking details about changes to patient management (if any). These might include changes to medications, mobilization of extra services, or regular monitoring.

After 3 months of regular reporting, general practitioners will be interviewed about their experiences, and the pooled data will be used in a final report.

## Results

At the time of writing we are running the data linkage process over 700,000 hospital presentations from a 10-year time frame and anticipate 100,000 unique patient records. We will then begin the process of stratifying the identified admissions into unavoidable, ambulatory sensitive, and other and perform the analysis. Following the analysis, we expect to provide a weighting to the various factors that will indicate risk of hospital admission and potentially a time frame. The combination will inform the general practitioners of the relative probability of hospital admission attributed to at-risk patients, thus allowing them to recommend appropriate interventions.

## Discussion

### Potential Implications

In the Australian context, this project is significant in two ways. In the first instance, the distributed nature of Australian general practice, with no formal registration to practices and split funding streams (general practice is federally funded and hospitals state funded), mitigates against quality data collection across the data silos. For that reason, the linking of data in these settings (a first for Australia) allows for investigations not previously possible. The second is the potential of delivering an almost real-time report to general practitioners to enable them to mobilize available resources to patients at the time. These resources may be from within the practice or from programs run by community or hospital services.

### Limitations

Data quality will always be a limitation in the data linkage process. The tool generation process is reliant on data quality from both the ED and the general practices. The ED dataset is derived from a set used to create the VEMD that is used for state-wide analysis and planning. It is collected by hospitals from their existing systems as a by-product of clinical and administrative processes. Similarly, the general practice data, while a more complete set, is also derived from data used for patient care. MEGPN has been involving practices in data quality and clinical governance reviews for 10 years; for certain fields the data are reliable and valid (prescribing, diagnoses) while for others (smoking status) the data are less reliable. This is one of the reasons data feedback loops are built into the program.

### Conclusions

With the agenda of keeping people out of hospital, the POLAR diversion project targets risk-of-presentation identification at general practice level. It aims to contribute meaningfully to the systematic, multifaceted approach to quality improvement that is inherent in good clinical governance and essential to best managing patients with complex problems.

By creating linkage between general practitioner and hospital records, we have been able to generate unique patient flow information. This will allow algorithms to be designed that will identify patients at risk of taking the less desirable care pathway via the local hospital ED, where resources are thinly spread. Design of a user-friendly report that can provide real-time data to primary care services will help direct patients to intervention services (eg, HARP, additional health care services), thus reducing the burden on the hospital system. By reducing ED traffic, patient outcomes are expected to improve via tailored care in a less acute environment.

## Abbreviations

ACSC: ambulatory care–sensitive conditions
CCDSS: computerized clinical decision support system
ED: emergency department
GRHANITE: Generic Health Network Information Technology for the Enterprise
HARP: Hospital Admission Risk Program
MEGPN: Melbourne Eastern General Practice Network
PARR++: Patients at Risk of Rehospitalization
POLAR: Population Level Analysis and Reporting
VEMD: Victorian Emergency Minimum Dataset
